# Cardiopulmonary exercise testing and impedance cardiography in the assessment of exercise capacity of patients with coronary artery disease early after myocardial revascularization

**DOI:** 10.1186/s13102-022-00527-w

**Published:** 2022-07-17

**Authors:** Małgorzata Kurpaska, Paweł Krzesiński, Grzegorz Gielerak, Karina Gołębiewska, Katarzyna Piotrowicz

**Affiliations:** Department of Cardiology and Internal Medicine, Military Institute of Medicine, ul. Szaserów 128, 04-141 Warsaw, Poland

**Keywords:** Hemodynamic parameters, Coronary artery disease, Exercise capacity, Cardiac rehabilitation

## Abstract

**Background:**

Patients with coronary artery disease (CAD) are characterized by different levels of physical capacity, which depends not only on the anatomical advancement of atherosclerosis, but also on the individual cardiovascular hemodynamic response to exercise. The aim of this study was evaluating the relationship between parameters of exercise capacity assessed via cardiopulmonary exercise testing (CPET) and impedance cardiography (ICG) hemodynamics in patients with CAD.

**Methods:**

Exercise capacity was assessed in 54 patients with CAD (41 men, aged 59.5 ± 8.6 years) within 6 weeks after revascularization by means of oxygen uptake (VO_2_), assessed via CPET, and hemodynamic parameters [heart rate (HR), stroke volume, cardiac output (CO), left cardiac work index (LCWi)], measured by ICG. Correlations between these parameters at anaerobic threshold (AT) and at the peak of exercise as well as their changes (Δpeak–rest, Δpeak–AT) were evaluated.

**Results:**

A large proportion of patients exhibited reduced exercise capacity, with 63% not reaching 80% of predicted peak VO_2_. Clinically relevant correlations were noted between the absolute peak values of VO_2_ versus HR, VO_2_ versus CO, and VO_2_ versus LCWi (R = 0.45, *p* = 0.0005; R = 0.33, *p* = 0.015; and R = 0.40, *p* = 0.003, respectively). There was no correlation between AT VO_2_ and hemodynamic parameters at the AT time point. Furthermore ΔVO_2_ (peak–AT) correlated with ΔHR (peak–AT), ΔCO (peak–AT) and ΔLCWi (peak–AT) (R = 0.52, *p* < 0.0001, R = 0.49, *p* = 0.0001; and R = 0.49, *p* = 0.0001, respectively). ΔVO_2_ (peak–rest) correlated with ΔHR (peak–rest), ΔCO (peak–rest), and ΔLCWi (peak–rest) (R = 0.47, *p* < 0.0001; R = 0.41, *p* = 0.002; and R = 0.43, *p* = 0.001, respectively).

**Conclusion:**

ICG is a reliable method of assessing the cardiovascular response to exercise in patients with CAD. Some ICG parameters show definite correlations with parameters of cardiovascular capacity of proven clinical utility, such as peak VO_2_.

## Introduction

Exercise capacity plays an important role in risk stratification in patients with coronary artery disease (CAD) [1, 2], as it is a potentially stronger predictor of mortality than other risk factors, such as smoking, hypertension, high cholesterol levels, and type 2 diabetes mellitus [3]. Moreover, patients with comparable exercise capacities have comparable mortality risks, irrespective of their baseline coronary revascularization status [4].

CAD patients may have various levels of exercise capacity, which is dependent not only on the severity of atherosclerosis but also on potential comorbidities and the individual cardiovascular hemodynamic response to exercise. The mechanism of exercise-induced increase in cardiac output (CO) may vary depending on heart rate (HR) and/or stroke volume (SV) alterations [5, 6]. Optimally, both components should increase simultaneously [6], with any deviations from this principle suggesting cardiovascular dysfunction. For instance, abnormal SV profile alterations during exercise in CAD patients who have undergone successful revascularization suggest microcirculatory dysfunction [7] and may affect the course and outcome of cardiac rehabilitation, which is related to the resting hemodynamic profile [8].

In everyday practice, the gold standard in noninvasive assessment of exercise capacity is cardiopulmonary exercise testing (CPET), which measures dynamic changes in such parameters as oxygen uptake (VO_2_), HR, and oxygen pulse (O_2_Pulse) [7, 9–11]. However, these parameters are only indirect indicators of hemodynamic adaptation to exercise. Moreover, these parameters may be confounded by concomitant respiratory and metabolic abnormalities. Impedance cardiography (ICG) is a novel noninvasive diagnostic technique for analyzing changes in CO and its components (HR and SV) during exercise testing. Preliminary research showed that ICG is a simple, accurate, and reproducible method of measuring these parameters over a wide range of workloads [12]. Our team’s previous studies [13, 14] demonstrated the usefulness of ICG in assessing the hemodynamic response to exercise in a group of hypertensive patients, by showing that ICG may complement traditional exercise testing and reveal an impaired hemodynamic response to exercise as a cause of unexplained dyspnea.

Therefore, the purpose of this study was to evaluate the relationship between the parameters of exercise capacity assessed via CPET and ICG hemodynamics in CAD patients who were candidates for cardiac rehabilitation.

## Methods

### Study group

The study enrolled 77 CAD patients of both sexes, aged 30–80 years, who were candidates for phase II cardiac rehabilitation, and had undergone coronary angioplasty or coronary artery bypass grafting within 6 weeks prior to recruitment. The exclusion criteria were significant coronary artery stenosis; confirmed secondary hypertension; chronic kidney disease with the estimated glomerular filtration rate (eGFR) < 30 mL/min/1.73 m^2^ calculated based on the MDRD equation; clinically important valvular heart disease; significant arrhythmias; non-sinus rhythm (including permanent pacemakers); body mass index (BMI) > 40 kg/m^2^; polyneuropathy; exercise-limiting peripheral artery disease and/or skeletal muscle disorders; psychiatric conditions preventing the patient’s full cooperation; and exacerbated lung conditions [asthma, chronic obstructive pulmonary disease (COPD)].

### Clinical examination

The clinical examination included past medical history; drug history; current symptoms, particularly exercise tolerance (fatigue, dyspnea on exertion and at rest, chest pain); and smoking status. Physical examination included office measurements of heart rate (HR), systolic blood pressure (SBP), and diastolic blood pressure (DBP), and anthropometric measurements (height, weight, BMI). Laboratory tests were conducted on fasting peripheral venous blood samples collected in the morning (7:00–8:30 a.m.), before CPET. The eGFR was calculated according to the MDRD equation.

### Echocardiography

Echocardiographic examinations were conducted with a Vivid S6 ultrasound system (GE Medical System, Wauwatosa, WI, USA) following revascularization and no more than 6 weeks prior to recruitment. The examination included standard parasternal, apical, and subcostal views, and assessed cardiac chamber size, valvular structure and function, left ventricular ejection fraction (LVEF) measured via the Simpson method, diastolic function, and evidence of left ventricular hypertrophy. Heart failure (HF) was diagnosed based on current guidelines [15].

### Cardiopulmonary exercise testing

Each patient underwent CPET in the morning, between 9:00 and 11:00 a.m., following the morning dose of their medications. An Ergoselect cycle ergometer (Geratherm Respiratory GmbH; Germany) was used, with individualized ramp protocols set to achieve the predicted load within 10 min. Oxygen and carbon dioxide sensors and the flow sensor (Ergoflow, Geratherm Respiratory GmbH; Germany) were calibrated before each test. Prior to CPET, each patient underwent resting spirometry. Each patient underwent maximum CPET, which was stopped if severe symptoms (fatigue, dyspnea) appeared or at the patient’s request [16]. Throughout each CPET session, breath-by-breath gas exchange was monitored via a Geratherm Ergostik system (Geratherm Respiratory GmbH; Germany). The following parameters were analyzed: VO_2_ [mL/kg/min], workload [W], O_2_Pulse [mL/min], relationship between oxygen uptake and work rate (VO_2_/WR) [mL/min/W], ventilatory efficiency (ventilation-to-carbon dioxide output, VE/VCO_2_slope) during exercise, respiratory exchange ratio (RER). These parameters were analyzed at rest prior to CPET, at the anaerobic threshold (AT) and at peakVO_2_. PeakVO_2_ was expressed as the highest mean oxygen consumption over the last 30 s of exercise. The AT was determined noninvasively, with the V-slope method after CPET conclusion [17]. Workload, VO_2_ and O_2_Pulse were expressed as percentage of their respective predicted values at peak exercise [peak % pred]. The predicted value of VO_2_ (pred VO_2_) was estimated based on Wasserman’s equation. [17]. VE/VCO_2_slope was calculated with a regression formula Microsoft Excel. O_2_Pulse was calculated as the quotient of VO_2_ and HR. The changes in VO_2_ (ΔVO_2_) between the value at rest and both at AT and at peak exercise (peak–rest, peak–AT) were calculated. The RER, defined as VCO_2_/VO_2_, represented the highest mean value from the last 30 s during the final stage of CPET.

### Exercise impedance cardiography

Exercise ICG was conducted with the use of a PhysioFlow monitor (Manatec, Paris, France). The methods employed during ICG were described in our earlier paper [13]. The device offered beat-to-beat acquisition of the following parameters: HR [bpm], SV [mL], CO [mL/min], and left cardiac work index (LCWi [kg∙m/m^2^]), with the last one defined as the estimated energy requirement of the left ventricle to eject blood against the aortic pressure. As it was done in the case of CPET parameters, we calculated the changes in these parameters between their values at rest and both at AT and peak exercise (peak–rest, peak–AT).

### Statistical analysis

Obtained results were analyzed statistically with Statistica 12.0 software (StatSoft Inc., Tulsa, OK, USA). Data distribution and normality were assessed visually and with the Kolmogorov–Smirnov test. Continuous variables were presented as means ± standard deviation (SD), whereas qualitative variables were presented as absolute and relative frequencies (percentages). Relationships between absolute LVEF, VO_2_, and hemodynamic parameters at AT and at peak exercise on one side and the changes in the analyzed exercise parameters (peak–rest, peak–AT) on the other were analyzed with Pearson/Spearman’s correlation coefficients. The *p* value of < 0.05 was considered statistically significant.

## Results

A total of 77 patients were included in the study. However, only the results of those 54 patients whose RER exceeded 1.05 (i.e. who completed maximum CPET) were ultimately analyzed. Their baseline characteristics are presented in Table 1. Nearly a half of the study group reported limited exercise tolerance, most commonly in the form of dyspnea. The vast majority of patients had preserved LVEF (mean LVEF of 54%), with 22.3% of patients diagnosed with HF. The most common concomitant conditions potentially affecting exercise tolerance were hypertension, smoking, and diabetes mellitus (reported by 72.2%, 53.7%, and 20.4% of patients, respectively). All patients were receiving optimal medical treatment for CAD.

**Table 1:** Baseline characteristics

Variable	Study group (n = 54)
Men, n (%)	41 (75.9)
Age (years), mean ± SD	59.5 ± 8.6
SBP (mmHg), mean ± SD	133.4 ± 18.6
DBP (mmHg), mean ± SD	77.5 ± 11.1
HR (bpm), mean ± SD	66.8 ± 11.9
BMI (kg/m^2^), mean ± SD	28.3 ± 4.1
Creatinine (mg/dL), mean ± SD	0.92 ± 0.20
eGFR < 60 mL/min/1.73 m^2^, n (%)	3 (5.6)
LVEF (%), mean ± SD	54.5 ± 7.8
LVEF < 50%,, n (%)	11 (20.4)
HFpEF, n (%)	3 (5.6)
HFmrEF, n (%)	4 (7.4)
HFrEF, n (%)	5 (9.3)
Hypertension, n (%)	39 (72.2)
Diabetes, n (%)	11 (20.4)
hypercholesterolemia, n (%)	45 (83.3)
COPD, n (%)	3 (5.6)
Atrial fibrillation paroxysmal in medical history, n (%)	5 (9.3)/0 (0.0)
Smoking, n (%)	29 (53.7)
Symptoms	
Reduced exercise tolerance, n (%)	25 (46.5)
Dyspnea during exercise, n (%)	14 (25.9)
Dyspnea at rest, n (%)	3 (5.6)
Pharmacotherapy	
ACEI, n (%)	53 (98.2)
ARB, n (%)	1 (1.9)
BB, n (%)	51 (94.4)
Diuretic, n (%)	20 (37.0)
CB, n (%)	11 (20.4)
MRA, n (%)	3 (5.6)
Statin, n (%)	53 (98.2)
Antiplatelet, n (%)	54 (100)

*ACEI* angiotensin converting enzyme inhibitor, *ARB* angiotensin receptor blocker, *BB* beta bloker, *BMI* body mass index, *CB* calcium canal blocker, *COPD* chronic obstructive pulmonary disease, *DBP* diastolic blood pressure, *eGFR* estimated glomerular filtration rate, *HFmrEF* heart failure with a mid-range ejection fraction, *HFpEF* heart failure with preserved ejection fraction, *HFrEF* heart failure with a reduced ejection fraction, *HR* heart rate, *LVEF* left ventricular ejection fraction, *LVH* left ventricular hypertrophy, *MRA* mineralocorticoid receptor antagonists, *SBP* systolic blood pressure, *SD* standard deviation

### Cardiopulmonary exercise test and impedance cardiography

Exercise capacity and hemodynamic parameters are presented in Table 2. Most patients exhibited low exercise capacity, with 63% of patients failing to exceed 80% of their predicted peak VO_2_. The VO_2_/WR values were generally low; however, VE/VCO_2_slope was normal in most patients. The increase in VO_2_ from AT to peak exercise was much lower than the increase in VO_2_ from its rest value to that at AT.

**Table 2:** Evaluation of exercise capacity and cardiovascular function via cardiopulmonary exercise testing with a simultaneous hemodynamic assessment via impedance cardiography

	Study group (n = 54)
CPET	
Peak workload (W), mean ± SD	129.4 ± 36.8
% pred. peak workload (%), mean ± SD	82.8 ± 12.9
VO_2_/WR (mL/min/W), mean ± SD	9.7 ± 1.5
VE/VCO_2_ slope, mean ± SD	28.7 ± 4.4
Rest VO_2_ (mL/min/kg), mean ± SD	3.6 ± 0.6
AT VO_2_ (mL/min/kg), mean ± SD	9.6 ± 2.5
Peak VO_2_(mL/min/kg), mean ± SD	18.2 ± 4.3
% pred. peak VO_2_ (%), mean ± SD	74.2 ± 13.5
% pred. peak VO_2_ < 80% VO_2_ of predicted value, n (%)	34 (63.0)
ΔVO_2_ (peak–rest) (mL/min/kg), mean ± SD	14.7 ± 4.1
ΔVO_2_ (peak–AT) (mL/min/kg), mean ± SD	8.7 ± 2.8
Rest O_2_pulse (mL/beat), mean ± SD	4.4 ± 0.9
AT O_2_pulse (mL/beat), mean ± SD	9.2 ± 2.3
Peak O_2_pulse (mL/beat), mean ± SD	12.4 ± 3.1
% pred. peak O_2_ pulse (%), mean ± SD	97.9 ± 23.0
ΔO_2_ pulse (peak–rest) (mL/beat), mean ± SD	8.0 ± 2.6
ΔO_2_ pulse (peak–AT) (mL/beat), mean ± SD	3.2 ± 1.4
ICG	
Rest HR (bpm), mean ± SD	67.7 ± 12.1
AT HR (bpm), mean ± SD	87.2 ± 14.1
Peak HR (bpm), mean ± SD	125.6 ± 18.3
ΔHR (peak–rest) (bpm), mean ± SD	57.9 ± 16.9
ΔHR (peak–AT) (bpm), mean ± SD	38.4 ± 16.8
Rest SV (mL), mean ± SD	80.1 ± 17.4
AT SV (mL), mean ± SD	99.7 ± 22.9
Peak SV (mL), mean ± SD	116.4 ± 27.6
ΔSV (peak–rest) (mL), mean ± SD	36.3 ± 21.9
ΔSV (peak–AT) (mL), mean ± SD	16.7 ± 19.6
Rest CO (L/min), mean ± SD	5.4 ± 1.2
AT CO (L/min), mean ± SD	8.7 ± 2.4
Peak CO (L/min), mean ± SD	14.5 ± 3.4
ΔCO (peak–rest) (L/min), mean ± SD	9.1 ± 3.1
ΔCO (peak–AT) (L/min), mean ± SD	5.8 ± 2.9
Rest LCWi (kg*m/m^2^), mean ± SD	3.3 ± 1.0
AT LCWi (kg*m/m^2^, mean ± SD	5.5 ± 2.0
Peak LCWi (kg*m/m^2^), mean ± SD	12.1 ± 4.2
ΔLCWi (peak–rest) (kg*m/m^2^), mean ± SD	8.8 ± 3.9
ΔLCWi (peak–AT) (kg*m/m^2^), mean ± SD	6.6 ± 3.5

*AT* value at anaerobic threshold; %pred. *AT* percentage of predictive value at anaerobic threshold, *peak* value at peak exercise; *%pred. peak* percentage of predictive peak value, *CO* cardiac output, *CPET* cardiopulmonary exercise test, *HR* heart rate, *ICG* impedance cardiography, *LCWi* left cardiac work index, *SD* standard deviation, *SV* stroke volume, *VE/VCO*_*2*_ ventilatory equivalent for carbon dioxide production, *VO*_*2*_ oxygen uptake, *WR* work rate, Δ changes in parameter between its measurements at rest, at peak exercise, and at the anaerobic threshold

**Fig. 1:**
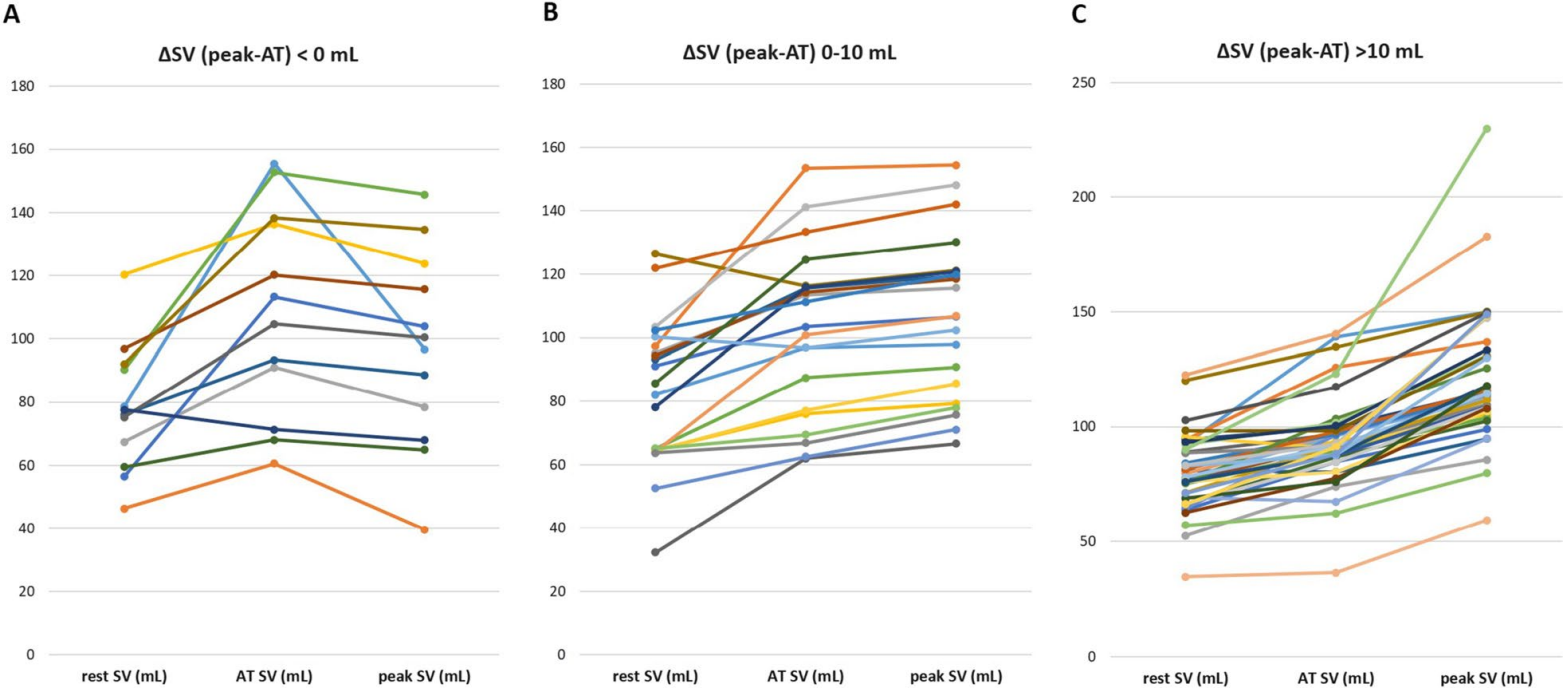
Individual trends of stroke volume: **A** subgroup with SV decrease between AT and peak of exercise, **B** subgroup with SV increase of 1–10 mL between AT and peak of exercise, **C** subgroup with SV increase over 10 mL between AT and peak of exercise

Throughout the exercise period (peak–rest), all patients demonstrated an increase in CO, both its components (SV and HR), and LCWi (Fig. 1). The increase in HR and CO that occurred after AT (peak–AT) constituted nearly a half of the total increase in these parameter values during the exercise period, whereas the increase in SV after AT was minimal. Conversely, LCWi increased more notably during the period after AT than at the beginning of exercise (AT–rest). As many as 12 patients (22.2%) showed a decrease in SV from the value at the AT to that at peak exercise, with a decrease in CO during the same period observed in 2 patients (3.7%). However, there were no cases of a decrease in HR between these time points.

The peak values of individual hemodynamic parameters were very widely distributed, with the broadest ranges observed for peak SV (median 114 mL; minimum 57% of median; maximum 201% of median), peak LCWi (11.8 kg∙m/m^2^; 40%; 197%, respectively), and peak VO_2_ (18.0 mL/min/kg; 39%; 168%, respectively). The distribution range was somewhat narrower for peak CO (median 14.4 mL/min; 58%; 153%, respectively), and it was the narrowest for peak HR (median 128 bpm; 67%; 130%, respectively).

### Correlations between VO_2_ values and those of selected hemodynamic parameters

Table 3 shows the correlations between absolute VO_2_ values and relative VO_2_ changes at various time points during exercise and the values of selected hemodynamic parameters. The evaluated group of patients demonstrated clinically relevant correlations between absolute values of peak VO_2_ and peak HR, peak VO_2_ and peak CO, and peakVO_2_ and peak LCWi. Furthermore, relevant correlations were observed between parameter values at the anaerobic point: VO_2_ and CO, VO_2_ and HR, and between VO_2_ and LCWi also.

**Table 3:** Correlations between the absolute values of VO_2_ at various CPET time points and those of selected hemodynamic parameters, as well as correlations between the relative changes in VO_2_ at various CPET time points and those in selected hemodynamic parameters

	HR (bpm)	SV (mL)	CO (L/min)	LCWi (kg*m/m^2^)
AT values				
VO_2_ (mL/min/kg)	0.32*	0.17	0.36***	0.33**
O_2_ pulse (mL/beat)	− 0.18	0.41**	0.26	− 0.02
Peak values				
VO_2_ (mL/min/kg)	0.45***	0.03	0.33*	0.40**
O_2_ pulse (mL/beat)	− 0.22	0.31*	0.19	− 0.01
Δvalues (peak–rest)				
VO_2_ (mL/min/kg)	0.47***	0.11	0.41**	0.43***
O_2_ pulse (mL/beat)	− 0.17	0.01	0.01	− 0.09
Δvalues (peak–AT)				
VO_2_ (mL/min/kg)	0.48***	0.03	0.41**	0.44***
O_2_ pulse (mL/beat)	− 0.21	0.08	− 0.05	− 0.25

*AT* anaerobic threshold, *CO* cardiac output, *HR* heart rate, *LCWi* left cardiac work index, *peak* value at peak exercise, *rest* value at rest, *SV* stroke volume, *VO*_*2*_ oxygen uptake, Δ changes in parameter between its measurements at rest, at peak exercise, and at the anaerobic threshold

^*^p < 0.05; ** p < 0.01; ***P ≤ 0.001

Changes in individual parameter values at specific time points showed the following correlations: ΔVO_2_ (peak–AT) showed correlations with ΔHR (peak–AT), ΔCO (peak–AT), and ΔLCWi (peak–AT) (R = 0.48, *p* = 0.0003; R = 0.41, *p* = 0.002; and R = 0.44, *p* = 0.0009, respectively); ΔVO_2_ (peak–rest) showed correlations with ΔHR (peak–rest), ΔCO (peak–rest), and ΔLCWi (peak– rest) (R = 0.47, *p* < 0.0001; R = 0.41, *p* = 0.002; and R = 0.43, *p* = 0.001, respectively). Neither the absolute SV values nor ΔSV showed any correlation with the corresponding absolute VO_2_ and ΔVO_2_ values in the total exercise period or in the period between AT and peak exercise. There was also no correlation between LVEF and peak VO_2_, peak SV, peak CO, or peak LCWi values.

### Correlations between O_2_pulse values and those of selected hemodynamic parameters

Clinically relevant correlations between absolute values of peak O_2_pulse and peak SV, O_2_pulse at AT and SV at was observed. There was no correlation between O_2_pulse values and other hemodynamic parameter values (HR, CO, LCWi) and between changes in O_2_pulse and changes in hemodynamic parameter values at specific time points.

## Discussion

Our study findings suggest that ICG can be useful in assessing individual cardiovascular hemodynamic response to exercise in CAD patients in the early post-revascularization period. Unlike CPET, ICG illustrates different patterns of change in the parameters that characterize the function of the heart as a pump. The changes in HR and SV contributed to a various extent towards an exercise-induced increase in CO, which may be clinically relevant.

The patients form this study group, who were assessed during early post-revascularization period, exhibited an exercise capacity (mean peak VO_2_ = 18.2 mL/min/kg) similar to that reported by Prado et al. [18] in patients after an acute coronary syndrome (18.8 mL/min/kg), but lower than that reported by Sparling et al. [19] in patients with stable CAD (22.9 mL/min/kg). Peak CO in our study group (14.5 L/min) was lower than that in healthy people (15–25 L/min) [20], but higher than that in patients with HF and low exercise tolerance (11.3 L/min) [21]. The increase in CO in our study group (9.1 mL/min) was comparable with that in patients with stable CAD (8.6 mL/min) [19] and higher than that in patients with HF with low exercise capacity (7 mL/min) [21]. Also the SV of 36.3 mL measured in our patients was higher than that in patients with HF and low exercise capacity (24 mL) [21].

The relationship observed between the hemodynamic parameters measured via ICG and VO_2_ supports the supposition that ICG may reveal hemodynamic causes behind limited exercise capacity. The observed increase in both absolute and percentage VO_2_ values was associated with an increase in both absolute and percentage CO values. The increase in both CO and HR was relatively constant throughout the period of exercise, whereas the increase in SV after AT was usually insubstantial; in fact, nearly one-third of patients showed a decrease in SV after AT, similar to Leprete's et al. observations [22]. The strongest correlations between the increase in VO_2_ and changes in hemodynamic parameters were observed during the intense workload period (peak–AT), which suggests that it is precisely during the later stage of exercise that the hemodynamic reaction affects the achieved peakVO_2_ most. Moreover, we would like to emphasize that it was precisely in the period between achieving AT and peak exercise that the interindividual variation in SV values was considerably greater than that in HR values (Fig. 1).

A relationship between low exercise capacity and the absence of SV increase during the final phase of the exercise test, as well as between low exercise capacity and low absolute SV values at peak exercise, have been reported in nonathletic adults [23] and in patients with hypertension [13], diastolic left ventricular dysfunction [24] and HF [12, 21]. Moreover, a number of authors have emphasized the association between regular physical exercise and SV improvement [25–27].

The decrease in SV during the final phase of the exercise test observed in 31.5% of patients may suggest a persisting myocardial dysfunction [7, 28]. Maintaining SV by patients with CAD is dependent mainly on the balance between preload, myocardial contractility, and afterload [6]. A clinically relevant aspect of myocardial ischemia is indicated by significant correlations between absolute SV values and O2 pulse at AT and at peak exercise. Goldkorn et al. demonstrated the correlation of early postexercise SV reduction with a extent of ischemia above 10% in myocardial perfusion imaging [29]. The abnormal SV reduction in response to exercise observed in the evaluated CAD patients post coronary revascularization, confirms Chaudhry’s [7] hypothesis of a significant association between peak VO_2_ and both microcirculatory function and peripheral factors. The effect of vascular stiffness on limiting exercise tolerance in CAD patients should be also considered while interpreting our findings [30]. Such an effect is indirectly implied by the significant correlation between CPET intensity and LCWi values, which are an indicator of the left ventricular capacity to work against the afterload.

If the mechanisms responsible for an adequate increase in SV fail (e.g. in the case of exercise-related myocardial ischemia), a compensatory chronotropic response is activated [7, 9–12, 28]. Consequently, HR becomes the component chiefly responsible for generating adequate CO during strenuous exercise. However, this comes at the expense of higher oxygen consumption [31]. In our study group it was also HR, and not SV, that showed a significant correlation with VO_2_. Nonetheless, a lack of linear correlation of selected hemodynamic parameters (particularly SV) does not rule out their clinical importance irrespective of VO_2_ values. Elucidating these issues requires further, well methodologically designed, studies. Our study demonstrated no correlation between LVEF and either VO_2_ or hemodynamic parameters, which is consistent with reports by other authors [32, 33].

We would like to emphasize the response to exercise in terms of LCWi, considered to be a strong predictor of cardiovascular risk in HF patients [34]. In our study group the mean peak LCWi was 12.1 kg*m/m^2^, which was a somewhat higher value than that reported by Myers et al. [34] in HF patients (10.4 kg*m/m^2^). The absolute peak LCWi value and changes in LCWi during exercise showed significant correlations with peak VO_2_ and ΔVO_2_, with the strength of these correlations comparable to that of the correlations observed between VO_2_ and CO. LCWi was distinct among the evaluated parameters, as it showed more dynamic changes during the peak–AT period. While interpreting these findings it is important to bear in mind that Lewicki et al. demonstrated a dynamic reaction of LCWi values to changes in the parameters that determine myocardial contractility [35]. This confirms the hypothesis that LCWi reflects the ability of the left ventricle to cope with the workload associated with increasing exercise intensity.

### Clinical implications

A noninvasive assessment of the hemodynamic profile during exercise in CAD patients provides additional, clinically important data on the factors that determine exercise capacity. Analysis of absolute values and trends in the changes of individual hemodynamic parameters may, in some cases, help identify the underlying causes of low exercise tolerance. Providing the possibility of assessing the profile of SV changes in addition to just assessing HR, the most commonly monitored parameter during exercise (e.g. sports training), may be useful in planning patient rehabilitation and identifying the causes of poor tolerance of training workloads. If CPET is unavailable, supplementing a traditional exercise test with ICG may be useful, with peak CO and LCWi values serving as indirect objective indicators of exercise capacity. The prognostic value of the parameters evaluated via ICG in this group of patients is still unknown; however, the results of our study encourage further research into this topic.

### Limitations

The limitations of our study were the relatively small sample size and patient heterogeneity resulting from various comorbidities (including heart failure with reduced and preserved EF) that may affect exercise capacity. Moreover, we assessed neither the patients’ pre-revascularization levels of exercise, nor the course of the coronary events preceding patient qualification for cardiac rehabilitation. Our assessments did not include any data on the level of patient motivation to complete the exercise test.

## Conclusions

Impedance cardiography is a reliable method of assessing the cardiovascular response to exercise in patients with coronary disease. Some ICG parameters show definite correlations with measures of cardiovascular capacity of proven clinical utility, such as peak VO_2_. Thanks to the possibility of monitoring the pattern of cardiovascular response to exercise, ICG may be useful in further research in this area.

## Data Availability

The datasets used and analyzed during the current study are available from the corresponding author on reasonable request. Data that supports the findings (demographic information and exercise test, ICG and other medical test results) were collected from medical records, and so are not publicly available. Data are however available from the authors upon reasonable request and with permission of Military Institute of Medicine.

## Abbreviations

AT: Anaerobic threshold
BMI: Body mass index
CAD: Coronary artery disease
CO: Cardiac output
COPD: Chronic obstructive pulmonary disease
CPET: Cardiopulmonary exercise testing
DBP: Diastolic blood pressure
eGFR: Estimated glomerular filtration rate
HF: Heart failure
HR: Heart rate
ICG: Impedance cardiography
LCWi: Left cardiac work index
LVEF: Left ventricular ejection fraction
O_2_Pulse: Oxygen pulse
peak %: Pred predicted values at peak exercise
pred VO_2_: Predicted value of VO_2_
RER: Respiratory exchange ratio
SBP: Systolic blood pressure
SV: Stroke volume
VE/VCO_2_slope: Ventilation-to-carbon dioxide output
VO_2_: Oxygen uptake
VO_2_/WR: Work rate
ΔVO_2_: Changes in oxygen output
